# On Specialties

**Published:** 1869-11

**Authors:** 


### *Specialties.*

Much comment is arising with regard to the more stringent enforcement of the rule against advertising of specialties, adopted by the American Medical Association. The letter of the law not infrequently seems repugnant to justice. It is a matter of notoriety that many of the most eminent members of the profession do restrict themselves to the practice of some particular specialty. They proclaim it in the colleges to which they happen to be attached, in the medical periodicals for which they write, the books they compile or edit, and, more loudly still, through partial friends in society.

It passes the comprehension of the present writer, why the briefer and more compendious mode of attaching the name of the specialty practiced to an ordinary business card or advertisement in the medical, or even secular press, should so horrify the brethren. A business card or advertisement, and a fulsome laudation of one's own skill and parade of certificates are very different affairs.

It would seem that the whole thing resolves itself to a mere question of taste, about which gentlemen may differ. To our mind, the specialist assumes the virtue of modesty, although he may have it not, when he writes, or prints his chosen function after his name, rather than, by the use of the major term only, giving space for the belief that he is ready for business in any department.

To the writer it appears that the real point is: Does the avowed specialist *know* any more about it than "any other man"?

